# [Retracted] MicroRNA-27a promotes tumorigenesis via targeting AKT in triple negative breast cancer

**DOI:** 10.3892/mmr.2026.14008

**Published:** 2026-09-03

**Authors:** Jing Wu, Zhihui Sun, Huijie Sun, Yanhua Li

Mol Med Rep 17: 562–570, 2018; DOI: 10.3892/mmr.2017.7886

Following the publication of the above paper, it was drawn to the Editor's attention by a concerned reader that certain of the cell migration and invasion data featured in Fig. 2C, G, H, K, L and O on p. 565-6 were strikingly similar to data which appeared in a handful of other articles written by different authors at different research institutes, one of which had already been published in *Journal of Biological Research-Thessaloniki* during the year before this paper was submitted to *Molecular Medicine Reports*.

Owing to the fact that the contentious data in the above article had already been published prior to its submission to *Molecular Medicine Reports*, the Editor has decided that this paper should be retracted from the Journal. The authors were asked for an explanation to account for these concerns, but the Editorial Office did not receive a reply. The Editor apologizes to the readership for any inconvenience caused.

